# Detection of *Lotmaria passim* and *Crithidia mellificae* in Selected Bumblebee Species

**DOI:** 10.3390/pathogens11091053

**Published:** 2022-09-15

**Authors:** Maria Michalczyk, Rajmund Sokół

**Affiliations:** Department of Parasitology and Invasive Diseases, Faculty of Veterinary Medicine, University of Warmia and Mazury in Olsztyn, Oczapowskiego 13, 10-719 Olsztyn, Poland

**Keywords:** bumblebee, *Lotmaria passim*, *Crithidia mellificae*, PCR, pathogens

## Abstract

Bumblebees (*Bombus* spp.) are an essential element of the ecosystem and the global economy. They are valued pollinators in many countries around the word. Unfortunately, there has been a decline in the bumblebee population, which is attributed to, among others, pathogens and reduced access to food due to the loss of natural nesting sites. *Lotmaria passim* and *Crithidia mellificae*, protozoan pathogens of the family Trypanosomatidae, commonly infect bumblebees, including in Poland. In this study, a Polish population of bumblebees was screened for *L. passim* and *C. mellificae*. The experiment was performed on 13 adult bumblebees belonging to 4 species: *B. lapidarius*, *B. lucorum*, *B. pascuorum*, and *B. terrestris*. Protozoa of the family Trypanosomatidae were identified by PCR. Only *L. passim* was identified in one *B. pascuorum* individual. Further research is needed to confirm the effect of concurrent pathogens on the decline of bumblebee populations.

## 1. Introduction

Bumblebees (*Bombus* spp.) are an essential element of the ecosystem and the global economy. They are valued pollinators in many countries of the word [1,2,3]. There are around 300 species of *Bombus* spp. in the world, 37 of which have been identified in Poland [4]. The most common species are *Bombus lapidaries*, *B. lucorum*, *B. pascuorum,* and *B. terrestris*. Bumblebee species are protected, but their populations continue to decline [5]. The exact cause of bumblebee mortality remains unknown, but it is believed that an increase in the number of pathogens may contribute to the decline of bumblebee populations [6,7,8]. Bumblebees are infected by protozoa of the family Trypanosomatidae, including *Lotmaria passim* and *Crithidia mellificae*. Trypanosomatids belong to the phylum *Euglenozoa*, class *Kinetoplastea*, subclass *Metakinetoplastina*, order *Trypanosomatida*, family *Trypanosomatidae* [9,10]. These protozoa were first detected in vertebrates and the honey bee [11,12]. Protozoa of the family Trypanosomatidae colonize the digestive system of honey bees [13,14]. They were also found in insects of the order *Hymenoptera*, bumblebees and mosquitoes [9,10,15,16,17,18]. Research has demonstrated that these pathogens are transmitted by the oral-fecal route [19]. There is evidence to indicate that trypanosomatids influence the health of insects by changing their lifestyle, lifespan, physiology, and immune response [20,21,22]. However, *L. passim* is currently considered the dominant protozoan globally [19,23,24,25,26]. On the other hand, the influence of *L. passim* and *C. mellificae* pathogens has not been thoroughly investigated to date. Currently, *L. passim* is most frequently detected in honey bees [19], whereas *C. mellificae* is identified sporadically [23]. The interest in trypanosomatids has significantly increased of late due to their possible role in bee mortality [27,28], high prevalence [23,25,29] and possible implication in honeybee colony losses [24,28,30]. Currently, *L. passim* is being studied due to its negative influence on the behavior, physiology, and immune response of bees [14,29,31]. In the authors’ previous research, *L. passim* and *C. mellificae* were detected in honey bees living in tree trunks, in bees kept in apiaries, and in the brood [26,32].

Bumblebees are highly effective pollinators, therefore their health should be closely monitored. Arismendi et al. [33] analyzed the presence of *Apicystis bombi, C. bombi*, and *Nosema bombi* viruses in bumblebees in southern Chile. They detected *A. bombi* and *C. bombi* (>78%) in *Bombus dahlbomii*, *Bombus terrestris,* and *Bombus ruderatus*. Multiple infections with *N. bombi, A. bombi,* and *C. bombi* were also reported in *B. terrestris* and *B. ruderatus.* The prevalence of *L. passim* was low (<6%) in bumblebee species. 

Gamboa et al. [34] also studied various pathogens in bumblebees. They analyzed the prevalence of ABPV, BQCV, DWV, LSV, SBV, *N. ceranae, C. bombi, Apicystis bombi,* and *Spiroplasma apis* in *B. stratus. Nosema ceranae* was omnipresent in the studied samples; *A. bombi* was detected in 12 out of 19 samples, and LSV was identified in 13 samples. *Nosema ceranae* was frequently detected in the tested samples, whereas *A. bombi* was noted in 12 out of 19 samples, and LSV - in 13 samples. In addition, Plischuk et al. [35] investigated the pathogens associated with *Bombus* spp. in Bolivia and Peru. They identified the AmFV virus, *N. ceranae, L. passim, C. bombi,* and five species of mites (of the genera *Parasitellus* Willmann, *Pneumolaelaps* Berlese, *Proctolaelaps* Berlese, *Tyrophagus* Oudemans, and *Kuzinia* Zachvatkinother) in bumblebees. *Crithidia bombi* was detected in 11 bumblebees, *L. passim* was observed in 7 out of 23 insects, and *V. ceranae* was identified in 4 bumblebees. They also detected various pathogens in pollinators. 

Pislak-Ocepek et al. [36] examined clinically healthy bumblebee (*Bombus* spp.) and honeybee workers to determine the incidence and prevalence of pathogens and to identify possible relationships between infections in bumblebees and honeybees. Insects were sourced from flowers at four different locations in Slovenia. Bumblebees were infected by ABPV (8.8%), BQCV (58.5%), DWV (6.8%), SBV (24.5%), LSV (15.6%), *N. bombi* (16.3%), *N. ceranae* (8.2%), *A. bombi* (15%), and *Crithidia bombi* (17%), whereas *N. apis* and *L. passim* infections were not observed. Their study confirmed that bumblebees can be simultaneously infected with several pathogens, many of which are shared with honeybees. In the present study *L. passim,* the most prevalent honeybee trypanosomatid, was identified in bumblebees. Similar observations were made by Bartolome et al. [37] who confirmed the simultaneous presence of *L. passim* and *C. mellificae* in two *B. terrestris* individuals.

Averill et al. [38] collected 1205 bumblebees from different flowering plants and found that all parasites were less prevalent in *Bombus impatiens* (69.2% parasite-free) than in *B. bimaculatus, B. perplexus*, and *B. vagans* (43.4% parasite-free). They also examined the prevalence of selected pathogens, including *L. passim*, in *Bombus* spp. Interestingly, trypanosomatid infections were significantly less prevalent in *B. impatiens* (11.0%) than in *B. vagans* (32.2%), *B. bimaculatus* (48.5%), or *B. perplexus* (49.5%). 

The aim of this study was to determine whether Polish bumblebee populations are infected with *L. passim* and *C. mellificae.* Little is known about the mechanisms responsible for the transmission of trypanosomes between different hosts, and the present study was undertaken to fill in this knowledge gap.

## 2. Results

**Figure 1:**
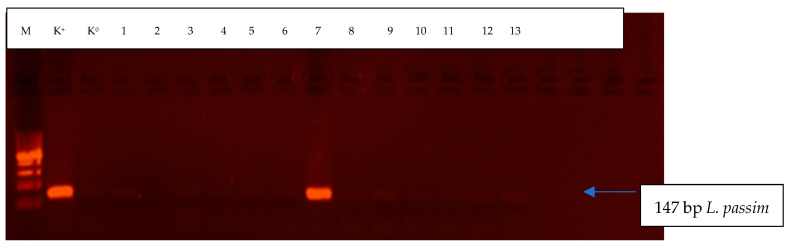
Electrophoretic separation of amplification products (*L. passim*) in bumblebee samples. M-GeneRuler^TM^ 100 bp DNA Ladder Plus volume marker (Fermentas); K^+^–positive control, includes DNA isolated from *L. passim* (University of Belgrade, Faculty of Veterinary Medicine, Department of Biology); K^0^–”zero” control, DNA was replaced with water; Paths 1 to 13–bumblebee samples.

A total of 13 bumblebees (samples) were analyzed. *Lotmaria passim* was identified in only one *B. pascuorum* individual. *Crithidia mellificae* was not found in any of the tested samples. Detailed results are presented in Table 1 and Figure 1.

## 3. Discussion

The latest molecular testing methods facilitate the search for and analysis of insect pathogens, including pathogens that cause asymptomatic infections. *Crithidia bombi* is most often detected in bumblebees [39], but the presence of *C. mellificae* has not been investigated to date. Therefore, the present study was undertaken to screen *Bombus* spp. for the presence of, among others, *C. mellificae.* The prevalence of protozoa of the family Trypanosomatidae, *L. passim* and *C. mellificae*, was low in 13 bumblebees from the Polish population. Only one sample tested positive for *L. passim*. *Crithidia mellificae* was not identified in any of the analyzed bumblebees. 

In the past, many pathogenic isolates had been identified as *C. mellificae* and were later reclassified to *L. passim*. At present, this species is considered to be the main pathogen of the honey bee [19]. Only *L. passim* was detected in the present study, which could be attributed to the fact that *L. passim* is much more prevalent in insects than *C. mellificae*. The prevalence of *L. passim* and *C. mellificae* in bees colonizing tree trunks was examined in our previous study. Twenty-six bees were sampled from tree trunks, and none of them showed clinical symptoms of parasitic infection. *Lotmaria passim* was identified in fourteen samples [26]. Bee larvae of different ages were also screened for parasites. *Lotmaria passim* was found in 21 colonies, but *C. mellificae* was not detected in any of the studied samples. Moreover, significant differences (*p* < 0.05) in the prevalence of *L. passim* were noted between 10-day-old larvae vs. 4-, 8-, 12-, 16- and 21- day-old bee brood [34], which suggests that these parasites are ubiquitous. Honeybees are often a source of infection for wild pollinators. According to Gajger et al. [40], honeybee viruses such as BQCV and DWV infect wild bumblebees in Croatia. Similar results were reported by Toplak et al. 2020 [41] who observed that honeybee viruses probably spill over from managed honeybee colonies to wild bumblebees that visit the same flowers. These observations were confirmed by Agler et al. [42] who found that the prevalence of DWV was higher in bumblebees collected near apiaries and when neighboring bees were highly infected. The prevalence of BQCV was also higher in bumblebees collected near apiaries. *Lotmaria passim* and *C. mellificae* could be transmitted between pollinators through flowers. In the present study, parasites were detected in bumblebees, and further research is needed to confirm that protozoa of the family Trypanosomatidae can be transmitted from honeybees to wild bumblebees, and that flowers can be an important route of transmission.

Further research is needed to determine the influence of *L. passim* and *C. mellificae* infections on the decline of bumblebee populations. The role played by these parasites in bumblebee populations should be investigated. In the present study, *L. passim* was also identified in pollinators.

## 4. Materials and Methods

Thirteen adult bumblebees belonging to 4 species (*B. lapidarius*, *B. lucorum*, *B. pascuorum,* and *B. terrestris*) were collected for the study. Bumblebees are protected in Poland, and the researchers received permission to catch only two bumblebees of each species (authorization from the Minister of the Environment, decision No. DLP-III-4102-327/2359/14/MD). Dead bumblebees (that died of natural causes without any disease symptoms) were harvested in 2015 in southern Poland. The insects were collected from a field in the Medicinal Herb Garden in Wrocław, Botanical Garden in Wroclaw, and the following landscape parks in Lower Silesia: Bystrzyca River Valley, Barycza River Valley, Jezierzyca River Valley, and Ślęża Mountain. Genomic DNA was isolated from whole bumblebees. Each insect was homogenized in a mortar under sterile conditions. The Genomic Mini kit (A&A Biotechnology, Gdynia, Polska) was used to isolate DNA. DNA was isolated according to the manufacturer’s instructions. The samples were stored in a freezer at −20 ℃ until further analysis.

The DNA isolated from *L. passim* and *C. mellificae* was identified with the use of HotStarTaq Plus Polymerase (Qiagen, Hilden, Germany) and the HotStarTaq Plus Master Mix Kit (Qiagen). The PCR mixture of 20 μL consisted of 10 μL of 2× HotStarTaq Plus Master Mix, 2 µL of CoralLoad Concentrate 10×, 0.1 μL of each primer, and 1 to 3 µL of DNA (around 1200 ng/reaction), supplemented with RNase-Free Water to 20 µL. The primers for the synthesis of *L. passim* and *C. mellificae* DNA [18] were developed by Genomed (Warsaw, Poland). The following primers were used: for *L. passim*–LpCytb_F2 5′-AGTaTGAGCaGTaGGtTTTaTTATa-3′ and LpCytb_R 5′-gcCAaAcACCaATaACtGGtACt-3; for *C. mellificae* –CmCytb_F 5′-AGTtTGAgCtGTtGGaTTTgTt-3 and CmCytb_R 5′-AACCtATtACaGGcACaGTTGC-3′ [18]. 

*Lotmaria passim* and *C. mellificae* were identified by single PCR under the following conditions: initial denaturation at 94 °C for 45 s, followed by 35 cycles at 94 °C for 45 s, primer annealing at 55 °C for 45 s, and elongation at 72 °C for 1 min. The final extension step was conducted at 72 °C for 10 min. Every PCR reaction had two controls: one positive control with *L. passim* or *C. mellificae* DNA (University of Belgrade, Faculty of Veterinary Medicine, Department of Biology) and one “zero” control where DNA was replaced with water. PCR products were separated by electrophoresis in 2% agarose gel containing 6 µL of the Midori green stain for visualizing the DNA fragments of *L. passim* and *C. mellificae.* Product size was compared with the GeneRuler_TM_ 100bp 36 Ladder Plus (Fermentas) molecular weight standard. Electrophoresis results were archived using the GelDoc (Bio-Rad, Hercules, CA, USA) imaging system. 

## Figures and Tables

**Table 1:** Presence of protozoa of the family Trypanosomatidae in bumblebee species.

Number of Samples	Bumblebee Species	*L. passim*	*C. mellificae*
1.	*B. lapidarius*	-	-
2.	*B. lapidarius*	-	-
3.	*B. lapidarius*	-	-
4.	*B. lapidarius*	-	-
5.	*B. lucorum*	-	-
6.	*B. pascuorum*	-	-
7.	*B. pascuorum*	+	-
8.	*B. pascuorum*	-	-
9.	*B. pascuorum*	-	-
10.	*B. pascuorum*	-	-
11.	*B. pascuorum*	-	-
12.	*B. terrestris*	-	-
13.	*B. terrestris*	-	-

## Data Availability

The data presented in this study are available on request from the corresponding author.
